# PML-II regulates ERK and AKT signal activation and IFNα-induced cell death

**DOI:** 10.1186/s12964-021-00756-5

**Published:** 2021-07-02

**Authors:** Xueqiong Meng, Yixiang Chen, Salvador Macip, Keith Leppard

**Affiliations:** 1 School of Basic Medicine, Henan University of Science and Technology, Luoyang, China; 2 Henan International Joint Laboratory of Thrombosis and Hemostasis, Luoyang, China; 3 Mechanisms of Cancer and Aging Laboratory, Department of Molecular and Cell Biology, University of Leicester, Leicester, UK; 4 FoodLab, Faculty of Health Sciences, Universitat Oberta de Catalunya, Barcelona, Spain; 5 School of Life Sciences, University of Warwick, Coventry, UK

**Keywords:** Promyelocytic leukemia protein, PML-II, IFNα, Apoptotic signaling, ERK, AKT

## Abstract

**Background:**

The requirement of promyelocytic leukaemia protein (PML) in interferon (IFN)-induced cell apoptosis is well-established. However, the exact mechanisms by which the multiple isoforms of PML protein participate in this process remain not well-understood. We previously demonstrated that PML isoform II (PML-II) positively regulates induced gene expression during a type I IFN response and evaluate here how PML-II contributes to IFNα-induced cell death.

**Methods:**

HeLa cells were transiently depleted of PML-II by siRNA treatment and the response of these cells to treatment with IFNα assessed by molecular assays of mRNA and proteins associated with IFN and apoptosis responses.

**Results:**

In HeLa cells, death during IFNα stimulation was reduced by prior PML-II depletion. PML-II removal also considerably decreased the induced expression of pro-apoptotic ISGs such as ISG54 (IFIT2), and substantially impaired or prevented expression of PUMA and TRAIL, proteins that are associated with the intrinsic and extrinsic apoptotic pathways respectively. Thirdly, PML-II depletion enhanced ERK and AKT pro-survival signaling activation suggesting that PML-II normally suppresses signaling via these pathways, and that lack of PML-II hence led to greater than normal activation of AKT signaling upon IFNα stimulation and consequently increased resistance to IFNα-induced apoptosis.

**Conclusions:**

The positive contribution of PML-II to the expression of various IFNα-induced pro-apoptotic proteins and its inhibition of pro-survival signaling together provide a mechanistic explanation for reduced apoptosis under conditions of PML deficiency and may account for at least part of the role of PML as a tumor suppressor gene.

**Video Abstract**

**Supplementary Information:**

The online version contains supplementary material available at 10.1186/s12964-021-00756-5.

## Background

Type I interferons (IFNα, β) are produced and secreted by cells in response to pathogen or damage sensing. They signal via cell surface receptors to activate expression of a large class of IFN-stimulated genes (ISGs), products from which act in multiple pathways to limit pathogen growth (reviewed in [1]). Whilst IFNα is thought to have, broadly, a cell survival effect on normal cells, protecting them from virus-induced apoptosis, it can induce apoptosis in tumour cells [2]. Indeed, IFNα has been used for the treatment of several types of haematological malignancies and solid tumors [3, 4].

Apoptosis can be triggered by either external or internally generated signals [5]. Several ISGs have been identified that have pro-apoptotic functions, for example ISG15 [6], ISG54 (IFIT2) [7, 8], XAF-1 (XIAP associated factor-1) & CD95 (Fas/APO-1) [9], and PML itself [10]. TRAIL/Apo2L (tumour necrosis factor-related apoptosis-inducing ligand) has also been demonstrated to be important for IFNα-mediated growth inhibition and apoptosis in cancer cells such as melanoma, myeloma and hepatocellular carcinoma [11–13]. Moreover, the expression of PUMA, an important pro-apoptotic protein of the intrinsic pathway, was also increased by IFNα stimulation in human myeloma cells [14]. As well as the action of specific ISGs, the inhibition of pro-survival signaling via the extracellular signal-regulated kinase (ERK) and phosphatidyl-inositol 3-kinase (PI3K)/AKT pathways is also an important mechanism for IFNα-mediated anti-tumour function. IFNα transiently diminished the phosphorylation of ERK in hepatocellular carcinoma cell lines suggesting an inhibition of this pathway [15]. IFNβ inhibition of AKT signaling was found to potentiate cisplatin-induced apoptosis in Hela cells [16] although PI3K signaling via mTOR was, in contrast, necessary for apoptosis induction in a multiple myeloma cell line [17].

Promyelocytic leukemia (PML) protein, an ISG product [18, 19], has multiple isoforms due to mRNA alternative splicing and post-translational modification, the regulation of which is not fully understood [20, 21]. The major isoforms are functionally distinct by virtue of their C-terminal domains, which recruit different interacting partners [22, 23]. The largest isoforms (PML-I and PML-II) are thought to be the most abundant, with other isoforms being very minor components in normal cells and more abundant in tumour cells, but still less than PML-I/II [24]. The tumour suppressive function of PML was first suggested by discovery of its disruption in a chromosome rearrangement that is characteristic of acute PML [25, 26]; the resulting fusion protein acts as a dominant-negative to block the activity of normal PML protein [10]. The tumour suppressive activity of PML was later extended to various solid tumours [27]. PML protein levels when compared to normal cells were found to be low in cancers including cervical, breast, lung and colon among others, correlating PML deficiency with tumorigenesis [28, 29], while loss of the *Pml* gene in a mouse model markedly accelerated tumour onset, incidence and progression [30]. It has been suggested that PML prevents cancer by inactivating nuclear AKT activity [30].

Several studies have shown that PML is required for efficient induction of apoptosis. Cells from PML-deficient mice showed severe apoptotic defects including a strongly decreased sensitivity to IFN-induced and death receptor-mediated apoptosis [10], while growth inhibition by IFNα in myeloma cells correlated with the presence of PML [11]; IFNα-induced apoptosis in hepatocellular carcinoma also involved PML [12]. In addition, cytoplasmic PML was required for apoptosis signaled by endoplasmic reticulum (ER) stress, opposing AKT survival signaling [31]. Lastly, the expression level of PML protein was shown to be closely related to the induction of cell death [32]. All these studies suggest that PML protein is an essential participant in or regulator of apoptosis induced by multiple routes including by IFN. However, the exact mechanism of this involvement remains to be completely understood. Among all the PML isoforms, PML isoform II (PML-II) is considered to be one of the most abundant isoforms suggesting a main contribution to PML functions [24, 33]. PML-II in particular potentiates the type I IFN response and ISG expression [34], suggesting that it may be an important player in IFN-mediated apoptosis. Here we show that loss of PML-II prior to IFNα stimulation results in reduced pro-apoptotic gene induction and increased cell survival signaling, correlating with a reduced efficiency of IFN-induced cell death.

## Materials and methods

### Cells and reagents

Human cervical cancer cell line, HeLa, was cultured in Dulbecco’s Modified Eagle Medium (DMEM) (Gibco) supplemented with 10% (v/v) foetal bovine serum (FBS) (Sigma) at 37 C in a 5% CO_2_ incubator. IFNα was from PBL Assay Science, poly(I:C) was bought from Sigma. PML, PML-I, PML-II, PML-V and control siRNA sequences (Additional File 1: Table S1) were synthesized by Ambion; Lipofectamine 2000 was purchased from Invitrogen.

### Flow cytometry

Cell death/apoptosis was determined by flow cytometry. Following 100 pmol/L siRNA transfection and IFNα or 1 μg/ml poly(I:C) stimulation at appropriate time points described, HeLa cells in 48-well cultures were released with trypsin and stained with 1 µg/m1of propidium iodide (PI) and incubated on ice for 20–30 min in the dark. After twice washing with cold PBS, cell staining was quantified using a FACSCanto II flow cytometer (BD Biosciences). Assays were performed in duplicate.

### SYBR-Green quantitative PCR

RNA was harvested from HeLa cell 24-well cultures using GenElute Mammalian Total RNA Miniprep Kit (Sigma-Aldrich) following the manufacturer’s instructions. Reverse transcriptions were performed using GoScript™ reverse transcriptase (Promega). Quantitative PCR (qPCR) reactions used SYBR-Green qPCR Master Mix and a Stratagene Mx3005P light cycler (Agilent Technologies). qPCR primers used in this study are listed in Supplementary Table 2. Data were analyzed using Agilent Technologies system software, with quantification based on Ct difference performed according to the “delta–delta *Ct* method” [35]. Target gene expression was normalized against expression of the housekeeping genes glyceraldehyde 3-phosphate dehydrogenase (GAPDH) or β-actin. All samples were analysed in triplicate.

### Western-blotting

Cells were lysed directly with SDS sample buffer (4% sodium dodecyl sulfate [SDS], 20% glycerol, 50 mM Tris HCl (pH 6.8)), proteins separated by SDS polyacrylamide gel electrophoresis and transferred to nitrocellulose membrane. Membranes were incubated overnight with specific primary antibodies diluted in blocking buffer. These included: TRAIL, PUMA, AKT, phospho-AKT(Ser473), phospho-p70S6K1(Thr389), ERKp44/p42, phospho-ERK(Thr202/Tyr204) and p-STAT1(Tyr701) from Cell Signaling Technology; MCL-1 and GAPDH from Santa Cruz Biotechnology; BCL-2 from DaKo; ISG15, ISG54, OAS1 and PML from Proteintech; β-actin from Millipore. After washing, bound antibodies were detected with fluorescent-conjugated secondary anti-rabbit or anti-mouse antibodies (Enzo Life Sciences), then visualized and quantified with an Odyssey system (Pierce, Waltham, MA, USA).

## Results

### PML-II regulates type I interferon-induced cell death

**Fig. 1:**
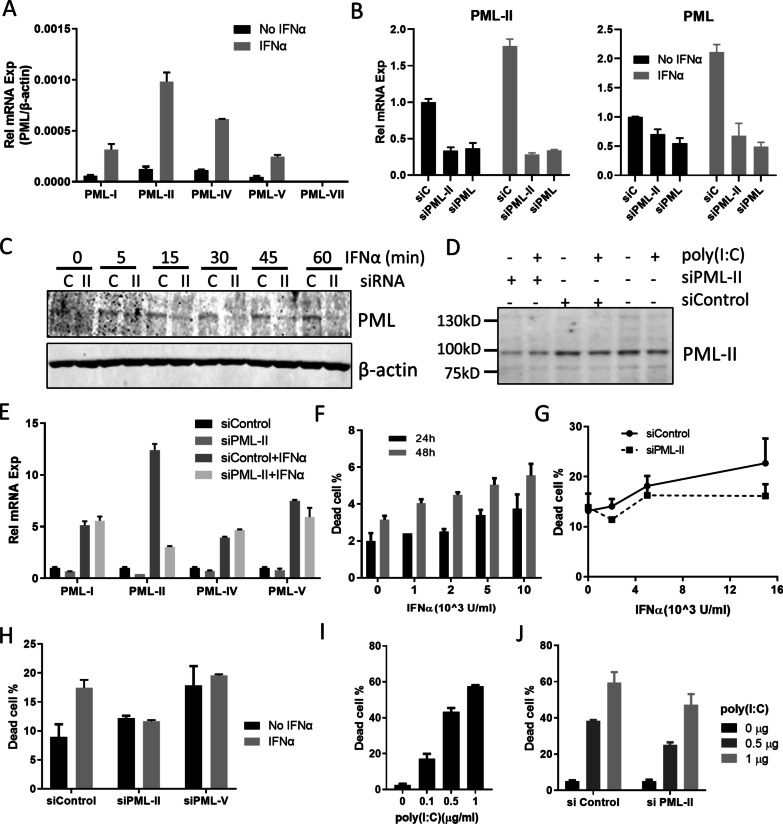
IFNα-induced cell death is PML-II dependent. **A** HeLa cells were stimulated with 1000 U/ml IFNα or not and PML isoform mRNA levels measured by SYBR-Green qPCR. The relative expression level of each PML isoform was quantified relative to β-actin. **B** 30–50% confluent HeLa cells were transfected with siPML-II, siPML or control siRNA for 48 h, and following 1000 U/ml IFNα or mock stimulation for 12 h, cells were collected and mRNA levels for PML-II and total PML were measured by SYBR-Green qPCR. The relative mRNA expression of each gene was quantified relative to GAPDH, and normalized to the level of unstimulated control siRNA-transfected cells. **C** HeLa cells, **D** HEK293 cells were transfected with PML-II or control siRNA for 48 h, then stimulated with 1000 U/ml IFNα at the described time points or 1 μg/ml poly(I:C) for 12 h. Total protein lysates were analyzed by western blotting for PML and PML-II, respectively. **E** Hela cells were transfected with either siPML-II or control siRNA for 48 h, and stimulated with 1000 U/ml IFNα for 12 h, then mRNA levels of PML-I, PML-II, PML-IV and PML-V were measured by SYBR-Green qPCR as in (**B**). **F** HeLa cells were treated with 1000 U/ml IFNα for 24 h or 48 h, and cell death quantified by PI staining and flow cytometry. **G** Hela cells were transfected with either siPML-II or control siRNA for 24 h, and following stimulation for 24 h with amounts of IFNα as indicated, cell death was quantified as in (**F**). **H** HeLa cells were transfected with either siPML-II or control siRNA for 12 h, then following 1000 U/ml IFNα stimulation for 48 h, cell death was detected as (**F**). **I** HeLa cells were transfected with the amounts of poly(I:C) indicated for 24 h, then cell death quantified as in (**F**). **J** HeLa cells were transfected with siRNA as in (**F**) for 24 h, then with 1 μg/ml poly(I:C) for 24 h and cell death quantified as in (**F**). Error bars show standard deviation among technical replicates

To investigate the biological role of PML-II in IFNα-induced cell death, we employed PML-II specific siRNA in HeLa cells, which are a cervical cancer cell line that has been shown to express PML-II and to be susceptible to IFNα-induced apoptosis [36, 37]; PML-II is a major PML isoform in these cells (Fig. 1A). Reflecting this abundance, siPML-II greatly inhibited PML-II expression but also detectably reduced total PML while PML siRNA (targeting all isoforms) not only inhibited PML expression but also decreased the expression of PML-II to a level similar to that achieved by PML-II siRNA (Fig. 1B). IFNα stimulation upregulated the expressions of all PML and PML-II specifically, and this upregulation was completely inhibited by either PML or PML-II siRNA (Fig. 1B). Reduced mRNA led to reduced PML protein (Fig. 1C, D) and, of the major isoforms, PML-II siRNA only had a significant effect on its expected target (Fig. 1E). Cell death increased with increasing dose and duration of exposure to IFNα (Fig. 1F). Importantly, knockdown of PML-II prior to IFNα treatment eliminated IFNα-induced cell death (Fig. 1G, H) whereas depletion of another isoform, PML-V, increased cell death independent of IFNα (Fig. 1H). We also tested the impact of PML-II depletion on cell death caused by poly(I:C), a synthetic analog of double-stranded RNA that is an effective type I IFN inducer [34, 38]. poly(I:C) stimulation quickly and effectively induced cell death in a dose-dependent pattern (Fig. 1I) and prior depletion of PML-II reduced this cell death response (Fig. 1J). These findings suggested that PML-II may have a general function regulating type I IFN-mediated apoptosis in HeLa cells that we investigated further.

### PML-II positively regulates IFNα-induced pro-apoptotic protein expression

**Fig. 2:**
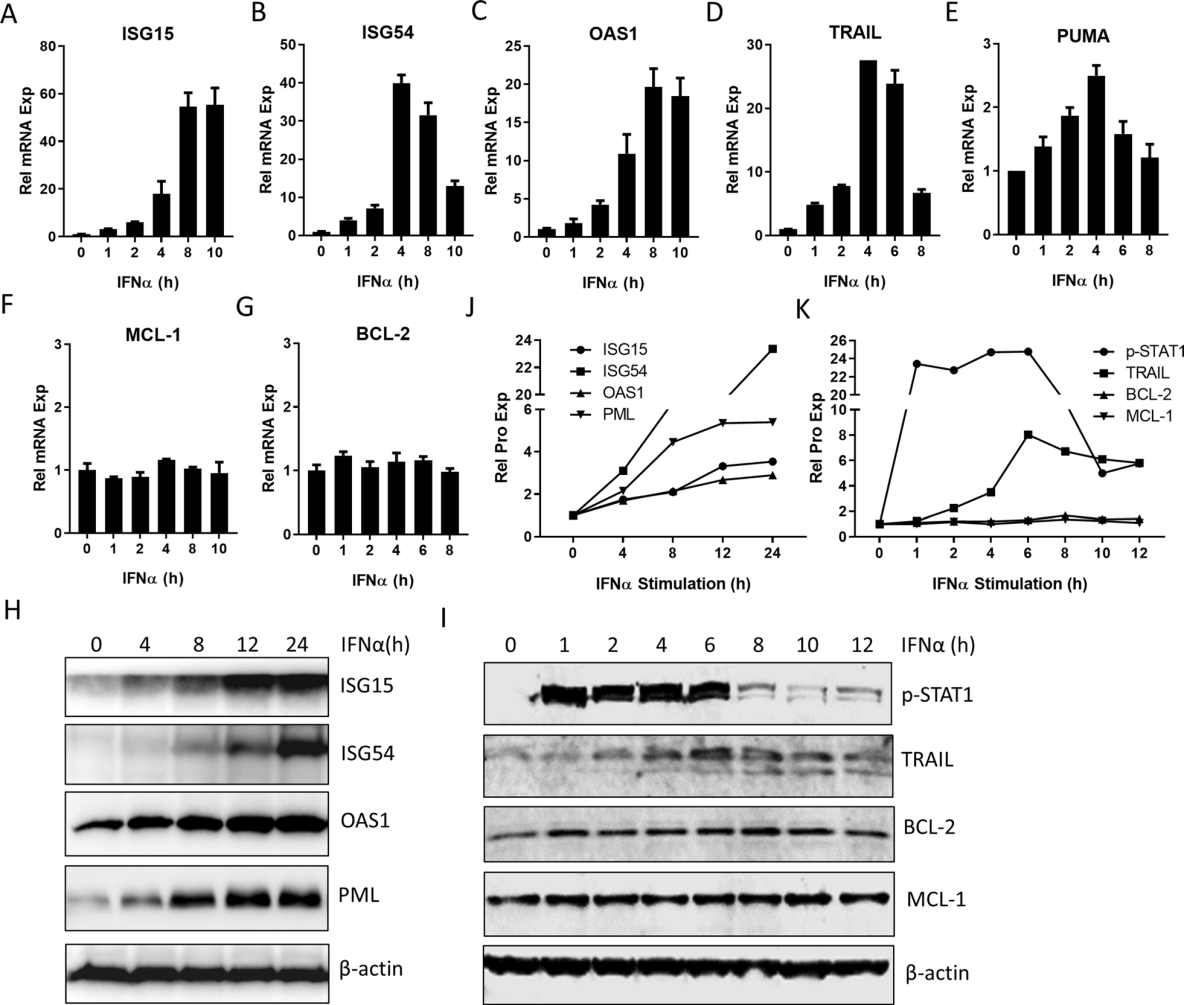
IFNα induces pro-apoptotic gene expression. HeLa cells were stimulated with 1000 U/ml IFNα and collected at different time points. (**A**–**F**) The mRNA levels of **A** ISG15, **B** ISG54, **C** OAS1, **D** TRAIL, **E** PUMA, **F** MCL-1 and **G** BCL-2 were measured by SYBR Green qPCR. The mRNA level of each gene was quantified relative to GADPH and normalized to the time zero value. Results are presented as mean ± SD of technical triplicate experiments. **H**–**I** Whole cell protein was analyzed by western blotting for ISG15, ISG54, OAS1, PML, p-STAT1, TRAIL, BCL-2 and MCL-1. β-actin was used as loading control. (**J**–**K**) The protein bands in panel H and I were visualized and quantified with an Odyssey system (Pierce), normalized to β-actin

**Fig. 3:**
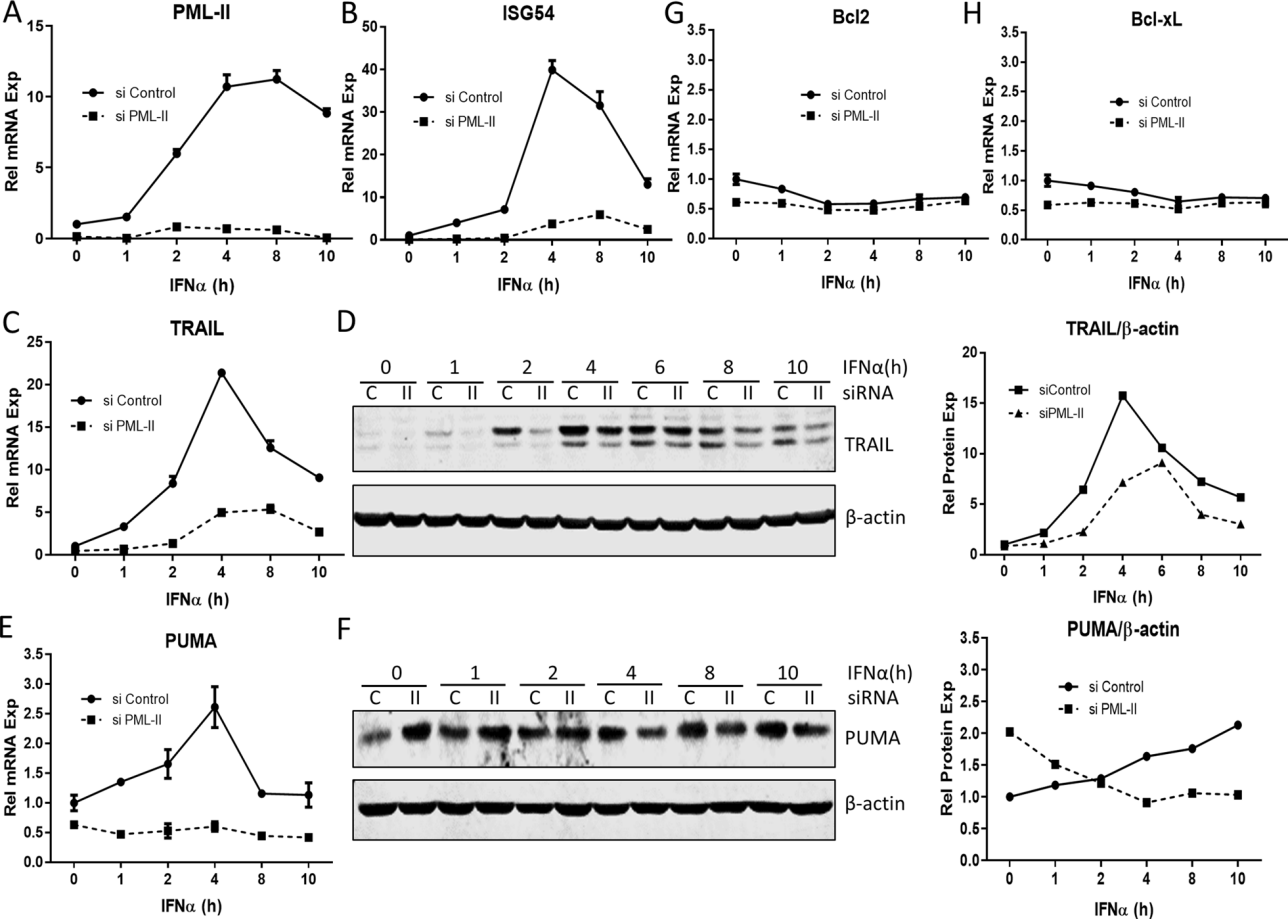
PML-II depletion downregulates pro-apoptotic gene expression. Hela cells were transfected with either PML-II siRNA or control siRNA for 48 h. Cells were stimulated with 1000 U/ml IFNα and were collected at described time points. (**A**–**C**, **E**, **G**, **H**) The mRNA expression levels of **A** PML-II, **B** ISG54, **C** TRAIL, **E** PUMA, **G** Bcl2 and **H** Bcl-xL were measured by SYBR Green qPCR. (**D**, **F**) The expression levels of **D** TRAIL and **F** PUMA proteins were detected by western blotting (left panels) and quantified by Odyssey system relative to β-actin (right panels). Error bars show standard deviation among technical replicates

As expected, expression of ISGs, including ISG15, ISG54 and OAS1, was greatly increased by IFNα (Fig. 2A–C). Expression of the death receptor TRAIL [13] and the pro-apoptotic gene PUMA was also rapidly induced by IFNα (Fig. 2D, E), although the scale of PUMA induction was much lower than for the ISGs and TRAIL. In contrast, IFNα stimulation had no effect on the expression of the anti-apoptotic BCL family members, MCL-1 (Fig. 2F) and BCL-2 (Fig. 2G). The impact of IFNα on TRAIL, ISG15, ISG54, OAS1, BCL-2 and MCL-1 was further confirmed at the protein level (Fig. 2H, I, quantified in Fig. 2J, K). We showed previously that IFNα-induced ISG mRNA levels were reduced substantially by prior depletion of PML-II [34]. As many of these products are pro-apoptotic [6, 8, 9], we next investigated the effect of PML-II depletion on apoptosis signaling components. Reduced expression of PML-II and ISG54 was achieved as expected (Fig. 3A, B). TRAIL induction was also greatly decreased at both the mRNA (Fig. 3C) and protein level (Fig. 3D) by PML-II depletion while the smaller scale induction of PUMA mRNA was abolished (Fig. 3E). The effect of PML-II on PUMA protein level was more complex (Fig. 3F). In uninduced cells, amounts of PUMA were two-fold greater in the absence of PML-II, suggesting increased stability since basal mRNA levels were if anything marginally reduced. Following IFNα stimulation, PUMA protein accumulated with similar kinetics to its mRNA in the presence of PML-II but in its absence not only was there no PUMA protein induction, in line with the lack of mRNA induction, but protein levels actually declined. In contrast, PML-II depletion had little effect on the expression of anti-apoptotic BCL family members BCL-2 and Bcl-xL (Fig. 3G, H).

### PML-II regulates ERK and AKT signaling

**Fig. 4:**
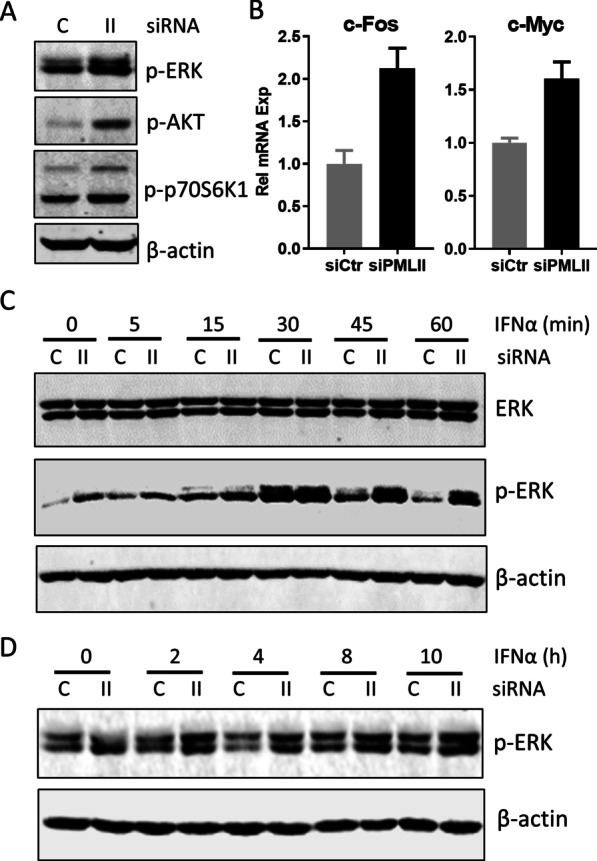
PML-II regulates ERK signaling. (**A**, **B**) HeLa cells were transfected with siPML-II or control siRNA, and after 48 h cells were collected. **A** Total protein lysates were analyzed by western blotting for p-ERK, p-AKT and p-p70S6K1. **B** Total cell RNA was analyzed by SYBR Green qPCR for c-Fos and c-Myc mRNA expression; amounts are expressed relative to GAPDH and normalized to the siControl sample. Error bars show standard deviation among technical replicates. **C**, **D** HeLa cells were transfected with siPML or control siRNA for 48 h, then stimulated with 1000 U/ml IFNα and collected at the described time points. Total protein lysates were analyzed by western blotting for ERK, p-ERK and p-AKT as indicated. Panel C is taken from the same experiment as Fig. 1C; the control β-actin data are duplicated for ease of reference

ERK and AKT signaling pathways link with proliferation and/or survival of various cancer cell lineages [39, 40]. We thus tested the effect of PML-II depletion on these pro-survival signals. Without any IFN stimulation, the phosphorylation level of ERK kinase (p-ERK) was increased by depleting PML-II (Fig. 4A), and the expression of ERK-dependent genes c-Fos and c-Myc was also upregulated (Fig. 4B), both indicating that PML-II has an inhibitory effect on ERK signaling. ERK protein levels were unchanged (Fig. 4C). Similarly, the levels of phospho-AKT (p-AKT) and phosphorylated p70S6K1, a downstream effector of AKT, were also enhanced by the depletion of PML-II (Fig. 4A), indicating that PML-II also inhibits this anti-apoptotic pathway.

Subsequently, we investigated the effect of PML-II on ERK signaling under the condition of IFNα stimulation. Upon IFNα addition to control cells, the level of phospho-ERK (p-ERK) began to increase within 5 min, peaked at 30 min and returned near to the basal level within one hour, and this low level of p-ERK was maintained in the following hours (Fig. 4C, D). Importantly, even under conditions of PML-II depletion, IFNα treatment still increased p-ERK levels further from the elevated basal level (Fig. 4C), suggesting an intrinsic inhibitory function of PML-II on ERK signaling, which was then further increased by IFNα stimulation independent of PML-II.

**Fig. 5:**
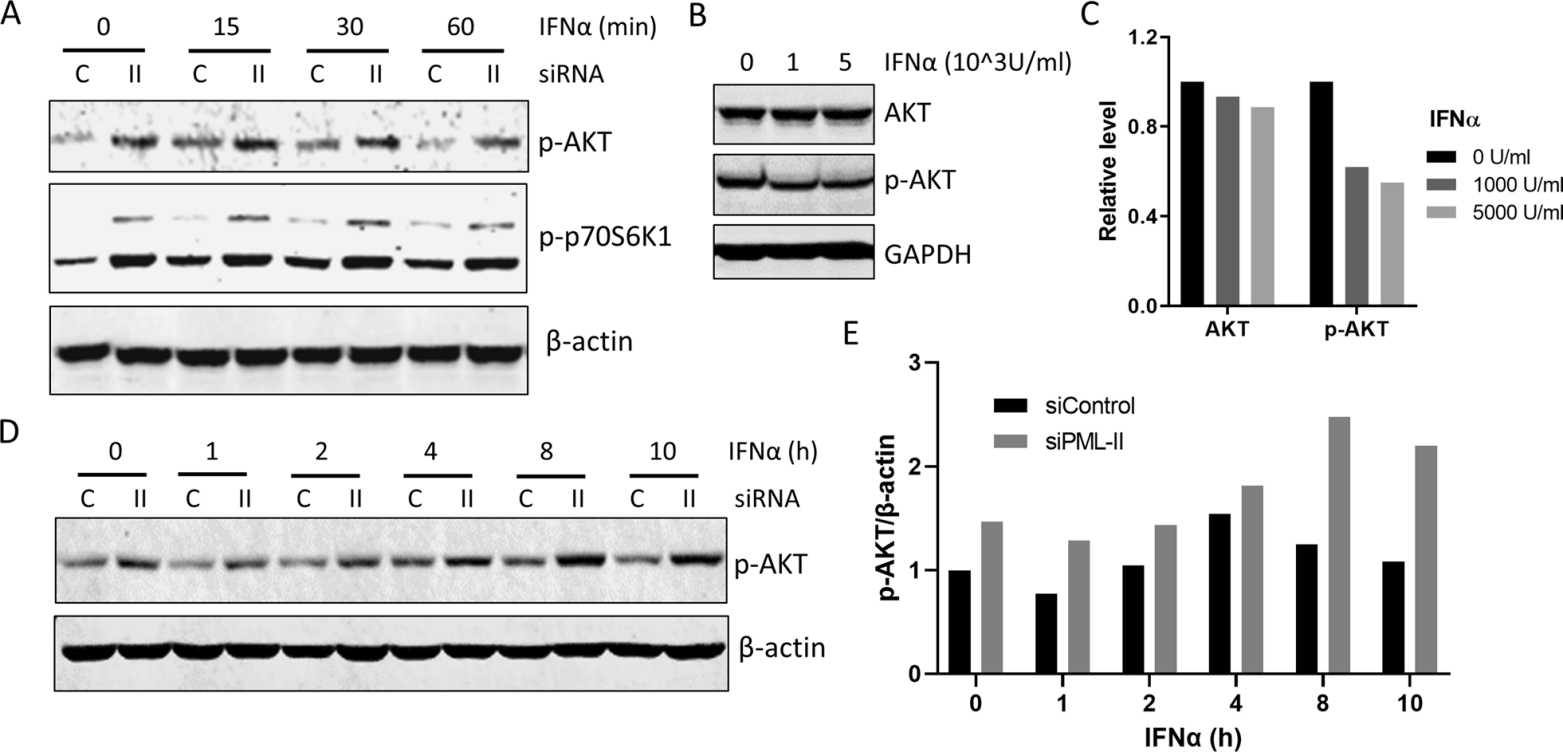
PML-II depletion increases AKT signaling. **A** HeLa cells were transfected and stimulated as Fig. 4C, and after the indicated times with IFNα, cells were lysed and the expression of p-AKT and p-p70S6K1 was detected by western blotting. **B** HeLa cells were stimulated with 1000 U/ml or 5000 U/ml IFNα for 72 h, then cells were lysed for WB testing appropriate proteins. **C** The graph shows the quantification of WB bands in (**B**) by using the Odyssey system (Pierce), normalized to β-actin and expressed relative to non-IFNα stimulation. **D** Cells were treated as Fig. 4C, and were collected at different time points for testing p-ATK by western blotting. **E** The bands of p-AKT in (**D**) were quantified by using the Odyssey system, values normalized to β-actin and expressed relative to control siRNA cells treated without IFNα stimulation

The short-term effect of IFNα on AKT signaling was similar to that on ERK. The level of p-AKT was elevated at 15 min post-stimulation, and then declined to the basal level at 60 min, and a similar result was also observed for phos-p70S6K1 (Fig. 5A). However, an inhibitory effect on p-AKT was observed after long-term IFNα stimulation (Fig. 5B, C). This long-term inhibition of the AKT pathway by IFNα could make an important contribution to IFNα-induced apoptosis. Finally, the role of PML-II in IFNα-regulated AKT signaling was determined (Fig. 5A, D). As observed for ERK, removing PML-II increased the base level of p-AKT. Some IFNα-induced drop in p-AKT occurred from this higher base level early post-stimulation, but the level rebounded by 8 h to amounts considerably higher than pre-stimulation (Fig. 5D, E). Altogether, these data demonstrated a similar pattern of effect of IFNα on AKT and ERK signaling in HeLa cells, with a transient increase and subsequent decrease in AKT and ERK signaling. PML-II repressed basal ERK and AKT signaling, limited the duration of stimulated ERK signaling during an IFN response, and inhibited longer term activation of the AKT pathway by IFNα signaling.

## Discussion

Previous work has established that PML is required for cell death. The data presented here show that the PML-II isoform specifically plays an important role in regulating cell death. Cell death following IFNα-stimulation was correlated with induction of pro-apoptotic factors such as TRAIL and PUMA, and with reduction in pro-survival signalling via AKT and ERK. When PML-II was depleted prior to IFNα treatment, both cell death and pro-apoptotic gene induction were reduced and the inhibition of pro-survival signalling normally produced by IFNα treatment was reversed. Because PML-II, being the product of an ISG, was itself strongly induced by IFNα treatment, its inhibition of pro-survival signalling will be further enhanced during an IFN response.

In this study, PML-II specific siRNA treatment not only greatly reduced PML-II but also significantly reduced total PML mRNA expression. This probably is because PML-II is one of the most expressed PML isoforms [24, 33]. The depletion of PML-II diminished cell death during IFN or poly(I:C) stimulation. This is consistent with the previous observations that PML protein is required for IFN-induced apoptosis [10, 32], and suggests that PML-II is one of the important isoforms involved in the process. HeLa cell death was observed following IFNα treatment in a dose- and a time-dependent pattern. The amount of dead cells was limited, probably because both the induction of pro-apoptotic proteins by IFNα and the reduction in pro-survival signalling were not sustained. Changes in expression level/activation level of these proteins peaked at 4–6 h after stimulation. HeLa cells also have multiple abnormalities and are relatively resistant to apoptosis because levels of p53 are kept low by the presence of human papillomavirus 18 E6 protein [41]. Moreover, cancer cells may respond differently to the different IFN subtypes. IFNβ is more potent compared to IFNα in inducing apoptosis in various cancer cells including melanoma, ovarian carcinoma and multiple myeloma cell lines [42–44]. This may explain why poly(I:C), an effective IFNα/β inducer, caused greater death in HeLa cells than IFNα alone.

During an IFN response, PML-II positively regulates the expression of pro-apoptotic ISGs [6, 8, 9]. Expression of TRAIL, which is the ligand for a death receptor and an ISG important for IFN-induced apoptosis in melanoma [11], was also found here to be strictly regulated by PML-II at both mRNA level and protein level, suggesting a role for PML-II in TRAIL death receptor-mediated apoptosis. This finding is consistent with the previous observation that loss of total PML decreased TRAIL expression in hepatocellular carcinoma cells [42] and correspondingly impaired IFNα-induced apoptosis [12]. In the present study, IFNα stimulation also induced expression of PUMA, an important effector in the mitochondria-mediated cell apoptosis pathway, and this induction was reduced by depleting PML-II. Overexpression of PUMA was found previously to cause rapid and profound apoptosis in colorectal cancer cells [45] and its level was increased by IFNα treatment in multiple myeloma [14]. In contrast to these IFNα and PML-II dependent increases in pro-apoptotic signaling components, the expression level of anti-apoptotic proteins of the BCL family was unaffected by IFNα stimulation. The involvement of PML-II in expression of both PUMA and TRAIL during IFNα-stimulation suggests that it is a positive regulator in both mitochondrial-mediated (PUMA) and death receptor-mediated (TRAIL) apoptotic pathways.

As well as inducing pro-apoptotic functions, type 1 IFN may exert anti-proliferative and pro-apoptosis activity by down-regulating survival signaling. The effects of IFNs on ERK and AKT signaling have been reported previously but with differing conclusions as to their activation [46–49] or suppression [15, 16, 50]. In the present study, we observed a transient activation of ERK and AKT signalling by IFNα in HeLa cells, however, when cells are exposed constitutively to IFNα, this situation is converted to a suppression of cancer cell survival, reflected in the long term inhibitory effect of IFNα on AKT signaling we observed.

PML-II was shown here to limit basal AKT activation and, during IFNα stimulation, PML-II depletion led to hyper-activation of AKT. PML (in total, not a specific isoform) is known to negatively regulate AKT activity by recruiting protein phosphatase 2A (PP2A) to PML-NBs, thereby dephosphorylating and inactivating AKT and loss of all PML species impairs PP2A, so increasing AKT activity [30]. Our findings suggest that PML-II is an important isoform for PP2A regulation and could exert its observed effect on AKT via this route. PML-II was also found to negatively regulate ERK signaling. Basal ERK signaling is strictly controlled by various negative regulators including PP2A, dual-specificity phosphatases (DUSP) and SPROUTY (SPRY) family proteins [51–53]. The negative effect of PML-II on the ERK pathway may, like the AKT pathway, also be determined by control of PP2A, or it may affect another of these known regulators. Further study is required to investigate these questions.

Reduced levels of PML protein have been observed in human cancers of multiple origins [27–29]. An increase in pro-survival signaling under low/absent PML-II conditions may be an important selective advantage for tumor growth. Several pathways that lead to increased turnover of all PML isoforms in tumour cells have been revealed [27], including ubiquitination by E6AP, an E3 ligase that is targeted in HPV-positive cervical carcinomas [54], and a hypoxia-induced mechanism mediated by KLHL20 [55]; proteasome inhibitor treatment promoted PML re-expression and restoration of PML-NBs in several PML negative tumor cell lines [28]. Recently, overexpression of PML was reported to inhibit cell growth and to significantly increase cell apoptosis in gastric cancer cells (56). In view of the role of PML-II in the regulation of IFN-mediated cell death, reduced or absent PML-II protein in tumors is also predicted to restrict the efficacy of IFNα anti-tumor activity.

## Conclusions

**Fig. 6:**
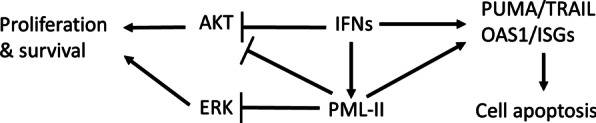
Regulation by PML-II of IFNα-induced cell death. IFN stimulation increases the expression of PML-II which in turn positively regulates IFNα-induced pro-apoptotic protein expression, contributing to IFNα-mediated anti-tumour activity. The intrinsic inhibitory effect of PML-II on AKT and ERK signaling also contributes to IFNα exerting this function

Collectively, both negative regulation of ERK and AKT signaling pathways by PML-II and the support PML-II provides for full induction of pro-apoptotic gene expression contribute to the growth suppressive effects of IFNα that depend on PML-II (Fig. 6). In light of the findings presented here, preventing PML-II degradation by targeting post-translational proteasome-dependent mechanisms of PML turnover, or increasing PML-II levels by other means, should sensitize cancer cells to IFN-induced cell death and provide a useful additional approach to therapy.

## Supplementary Information

**Additional file 1**. siRNA and qPCR primer sequences used in the study.

## Data Availability

All data generated or analysed during this study are included in this published article [and its Additional files].

## Abbreviations

IFN: Interferon
ISG: Interferon-stimulated gene
TRAIL: Tumour necrosis factor-related apoptosis-inducing ligand
ERK: Extracellular signal-regulated kinase
PI3K: Phosphatidyl-inositol 3-kinase
AKT: Protein kinase B
PML: Promyelocytic leukemia
PML-II: Protein isoform 2 from the *PML* gene
ER: Endoplasmic reticulum
PI: Propidium iodide
GAPDH: Glyceraldehyde 3-phosphate dehydrogenase
p-AKT: Phosphorylated AKT
p-ERK: Phosphorylated ERK
p-STAT1: Phosphorylated signal transducer and activator of transcription 1
siPML-II: Short interfering RNA targeting PML-II mRNA
