# Supplementary material for: PML-II regulates ERK and AKT signal activation and IFNα-induced cell death
Source: Cell Commun Signal. 2021 Jul 2;19:70. doi: 10.1186/s12964-021-00756-5 (PMC8252201; doi:10.1186/s12964-021-00756-5)
Supplement: Supplementary file 2 — Additional file 1. siRNA and qPCR primer sequences used in the study. [file 12964_2021_756_MOESM2_ESM.docx]

Supplementary table 1 siRNA sequences (Chen et al., 2015; Wright, 2010)

| **siRNA** | **Sense 5’-3’** | **Antisense 5’-3’** | **Description** |
| --- | --- | --- | --- |
| Control siRNA | GAGCCGGACGCCAAAGAAAUU | UUUCUUUGGCGUCCGGCUCUU | Negative control no sequence targeting any *Homo sapiens* coding sequence, |
| siPML | GAGCUCAAGUGCGACAUCAUU | UGAUGUCGCACUUGAGCUCUU | Specific targeting exon 3 of PML mRNA |
| siPML-I | CGUGAGCUUCAUGGAGCUGUU | CAGCUCCAUGAAGCUCACGUU | Specific targeting exon 9 of PML I mRNA |
| siPML-II | CAUCCUGCCCAGCUGCAAAUU | UUUGCAGCUGGGCAGGAUGUU | Specific targeting exon 7b of PML-II mRNA |
| siPML-V | GUUCAGCCCAGGACUCCUGUU | CAGGAGUCCUGGGCUGAACUU | Specific targeting exon 7 of PML V mRNA |

Supplementary table 2 qPCR primer

| **Gene** | **Forward 5'−>3'** | **Reverse 5'−>3'** | **Reference** |
| --- | --- | --- | --- |
| PML-I | CAGCATCTACTCCAAGGCCG | GTAGCAGGCCAAGATAGGGC | This paper |
| PML-II | AGGCAGAGGAACGCGTTGT | GGCTCCATGCACGAGTTTTC | (Chen et al., 2015) |
| PML-IV | TGGACGAGAACCTTGCTGAC | CCCCAGGAGAACCCACTTTC | This paper |
| PML-V | GGGAGGCAGAGGAACGC | CTGAGAGTCACCTGCTGTGG | This paper |
| PML-VII | TATTGACGTTGACCTGCTGCC | ATGCATAGCCATTCATTCGTTGA | This paper |
| PML | CGCCCTGGATAACGTCTTTTT | CTCGCACTCAAAGCACCAGA | (Wang et al., 2012) |
| ISG15 | CCCTCGAAGGTCAGCCAGA | GGACAAATGCGACGAACCTCT | (Han et al., 2011) |
| ISG54 | TGCAACCTACTGGCCTATCTA | CAGGTGACCAGACTTCTGATT | (Shi et al., 2010) |
| c-Fos | CCGGGGATAGCCTCTCTTACT | CCAGGTCCGTGCAGAAGTC | (Wang et al., 2012) |
| c-Myc | GGCTCCTGGCAAAAGGTCA | CTGCGTAGTTGTGCTGATGT | (Wang et al., 2012) |
| PUMA | GACCTCAACGCACAGTACGAG | AGGAGTCCCATGATGAGATTGT | (Wang et al., 2012) |
| TRAIL | TGCGTGCTGATCGTGATCTTC | GCTCGTTGGTAAAGTACACGTA | (Wang et al., 2012) |
| OAS1 | TGCGCTCAGCTTCGTACTGA | GGTGGAGTTCTCGCCCTCTT | (Wang et al., 2012) |
| BCL-2 | GGTGGGGTCATGTGTGTGG | CGGTTCAGGTACTCAGTCATCC | (Wang et al., 2012) |
| BCL-xL | GAGCTGGTGGTTGACTTTCTC | TCCATCTCCGATTCAGTCCCT | (Wang et al., 2012) |
| MCL-1 | GTGCCTTTGTGGCTAAACACT | AGTCCCGTTTTGTCCTTACGA | (Wang et al., 2012) |
| GAPDH | GGTCGGAGTCAACGGATTT | CCAGCATCGCCCCACTTG | (Vestergaard et al., 2011) |

**Reference**

Chen, Y., Wright, J., Meng, X., and Leppard, K. N. (2015). Promyelocytic Leukemia Protein Isoform II Promotes Transcription Factor Recruitment To Activate Interferon Beta and Interferon-Responsive Gene Expression. Mol Cell Biol *35*, 1660-1672.

Han, Q., Zhang, C., Zhang, J., and Tian, Z. (2011). Involvement of activation of PKR in HBx-siRNA-mediated innate immune effects on HBV inhibition. PLoS One *6*, e27931.

Shi, H. X., Yang, K., Liu, X., Liu, X. Y., Wei, B., Shan, Y. F., Zhu, L. H., and Wang, C. (2010). Positive regulation of interferon regulatory factor 3 activation by Herc5 via ISG15 modification. Mol Cell Biol *30*, 2424-2436.

Vestergaard, A. L., Knudsen, U. B., Munk, T., Rosbach, H., and Martensen, P. M. (2011). Transcriptional expression of type-I interferon response genes and stability of housekeeping genes in the human endometrium and endometriosis. Mol Hum Reprod *17*, 243-254.

Wang, X., Spandidos, A., Wang, H., and Seed, B. (2012). PrimerBank: a PCR primer database for quantitative gene expression analysis, 2012 update. Nucleic Acids Res *40*, D1144-1149.

Wright, J., PhD Thesis, University of Warwick (2010). The role of PML proteins in adenovirus type 5 infection and the type I interferon response.
